# Evaluation of Resection Margin after Image-Guided Dural Tail Resection in Convexity Meningiomas

**DOI:** 10.3390/jcm10061177

**Published:** 2021-03-11

**Authors:** Darius Kalasauskas, Yasemin Tanyildizi, Mirjam Renovanz, Marc A. Brockmann, Clemens J. Sommer, Florian Ringel, Naureen Keric

**Affiliations:** 1 Department of Neurosurgery, University Medical Centre, Johannes Gutenberg University Mainz, 55131 Mainz, Germany; mirjam.renovanz@med.uni-tuebingen.de (M.R.); florian.ringel@unimedizin-mainz.de (F.R.); naureen.keric@unimedizin-mainz.de (N.K.); 2 Department of Neuroradiology, University Medical Centre, Johannes Gutenberg University Mainz, 55131 Mainz, Germany; yasemin.tanyildizi@unimedizin-mainz.de (Y.T.); marc.brockmann@uni-mainz.de (M.A.B.); 3 Department of Neurosurgery, University Hospital Tübingen, Eberhard Karls University Tübingen, 72076 Tübingen, Germany; 4 Department of Neurology & Interdisciplinary Neuro-Oncology, University Hospital Tuebingen, Hertie Institute for Clinical Brain Research, 72076 Tübingen, Germany; 5 Institute of Neuropathology, University Medical Centre, Johannes Gutenberg University Mainz, 55131 Mainz, Germany; Clemens.Sommer@unimedizin-mainz.de

**Keywords:** meningioma, dural tail, resection margin, navigation, radiomics

## Abstract

Infiltration of adjacent dura with meningioma cells is a common phenomenon. Wide resection of the dural tail (DT) to achieve a gross total resection is a general recommendation. We aimed to investigate a tumor cell infiltration of the DT after image-guided resection of convexity meningiomas. The study’s inclusion criteria were the diagnosis of convexity meningioma, planned Simpson I° resection, and an identifiable DT. Intraoperative image-guidance was applied to identify the outer edge of the DT and to guide resection. After resection, en-bloc specimen or four samples of outermost pieces of DT in case of piecemeal resection were sent for histological analysis. In addition to resection margin infiltration, the radiological extent of DT, radiomic characteristics (109 in total), histology, and demographic data were assessed. Hierarchical clustering was used to generate patient clusters for radiomic analysis. Twenty-two patients were included in the study, while 20 (91%) were female. The mean age was 54.2 (Standard deviation (SD) 13.9, range 30–85) years. En-bloc resection could be achieved in 4 patients. The remaining patients received piecemeal resection. 2 DT samples were omitted due to tumor infiltration of the superior sagittal sinus. None of the en-bloc resection samples demonstrated dural infiltration on the resection margin. Tumor cells were detected in 4 of 70 (5.7%) dural tail samples and could not be excluded in another 5 of 70 (7.1%). No tumor recurrences were detected at follow-up MRI examinations after a mean follow-up of 27.5 (SD 13.2, range 0 to 50.0) months. There was no significant association between DT infiltration and histological subtype or patient characteristics and between DT extent and tumor infiltration. Clustering according to radiomic characteristics was not associated with tumor infiltration (*p* = 0.89). The radiological dural tail does not reliably outline the extent of tumor cell infiltration in convexity meningiomas. Hence, the extent of dural tail resection should not exclusively be guided by preoperative radiological appearance.

## 1. Introduction

Meningioma is the most common primary intracranial tumor, arising from non-neuroepithelial tissue, arachnoid cap (meningothelial) progenitor cells [1] and comprising up to one-fourth to one-third of all intracranial neoplasms [2]. The dural tail (DT) sign, first described in 1989, is a contrast-enhancing thickening of dura that is contiguous with the meningioma’s dural attachment and is seen on T1-weighted magnetic resonance imaging (MRI) sequences [3]. Among intracranial tumors, DT is most characteristic for meningiomas [4] and is seen in more than half of cases [5], although it is not specific and may be found in metastases, glial tumors, solitary fibrous tumors/hemangiopericytomas, extra-axial sarcoidosis, and certain other conditions (reviewed in detail in [6]) as well. 

The significance of the DT and the mechanism of its development are not yet fully understood. It was initially suggested that the thickening of the dura represents an infiltration of the dura with meningioma tumor cells [3]. However, further investigations revealed that it most probably arises through reactive changes such as hypervascularity, and dilation of blood vessels, as tumor cells invade and proliferate at the point of dural attachment, thus explaining a more significant contrast enhancement in the tail as in the tumor [7,8].

**Figure 1:**
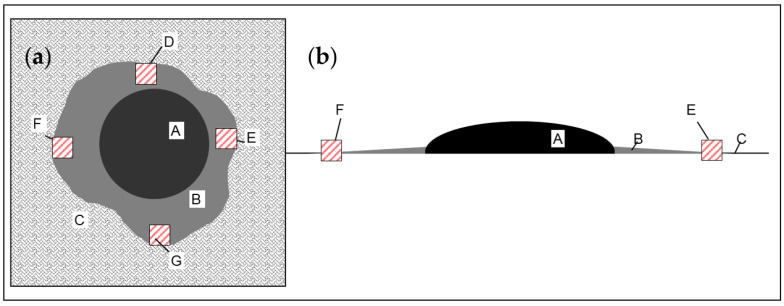
Schematic figure of a tumor and dural tail resection. (**a**): view from above. (**b**): view from the side. A—meningioma, B—dural tail, C—normal dura, D–F—separately resected dural specimens for histological analysis.

As with every tumor, the usual aim of meningioma resection is to achieve maximally safe and complete removal of the tumor according to a Simpson grade 1 resection, including the dural attachment. However, it is not known whether the extent of DT should guide the surgeon. Tumor infiltration of the DT has been reported in 35% and 88% of samples (Figure 1) [9,10,11,12]. But the exact amount of dura necessary to be resected to achieve a significant difference in tumor-free survival is disputed. Resection of the DT or surrounding dura up to 2.5 cm was proposed [8,9,10], which may lead to extensive craniotomies and dural reconstruction. Furthermore, there are no radiological criteria that would help to identify tumor-infiltrated parts of DT. However, even after gross total resection of World Health Organization (WHO) grade I meningiomas, tumor recurrence may occur, with an estimated 5-year progression-free survival slightly over 95% [13,14].

Thus, it remains unclear whether the resection should include DT to achieve tumor-free resection borders. In this prospective study, we investigated the infiltration of the distal DT after image-guided resection of convexity meningiomas.

## 2. Patients and Methods

### 2.1. Patient Data

**Figure 2:**
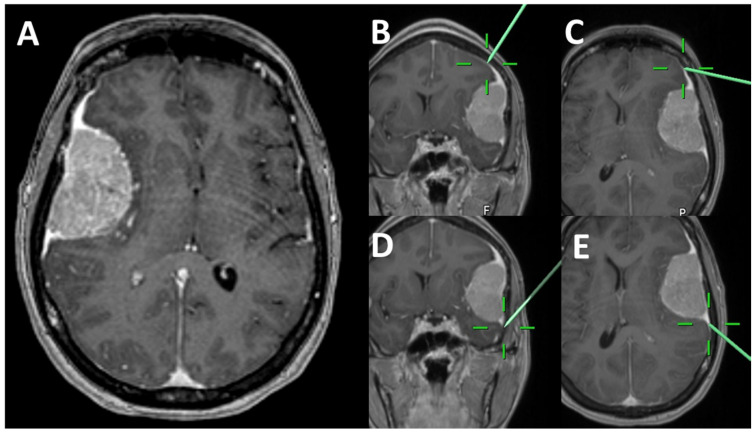
Illustrative image demonstrating intraoperative screenshots demonstrating the localization of dural tail specimens. (**A**)—preoperative MRI demonstrating a convexity meningioma with a dural tail. (**B**–**E**)—intraoperative screenshots of the locations of dural tail specimens. MRI, magnetic resonance imaging.

Patients in our department planned for Simpson grade I [15] (see Table S1 for grading) resection of a convexity meningioma with an identifiable DT were eligible for inclusion in the study. The patients without identifiable DT, wide attachment to falx or skull base or extradural extent of meningioma were excluded. The study included consecutive convexity meningiomas operated between 2015–2017, and several inconsecutively recruited patients until 2020. Intraoperative image-guidance was prepared before the operation using standard thin-sliced contrast-enhanced T1 MRI sequences and Brainlab iPlan (Brainlab, Heimstetten, Germany) navigation software. The surgeon´s estimation confirmed the complete removal of the DT. There were two approaches for examination of the infiltration of the outermost portion of DT. In cases where en-bloc resection of meningioma and DT was possible, the histological examination of DT’s complete outer part was performed. If en-bloc resection of the meningioma with the entire DT was not possible, the outermost portions of the resected DT were excised (rostral, dorsal, medial, and lateral, Figure 1) and sent for histological analysis with a focus on tumor infiltration. Intraoperatively, screenshots were obtained for comparison with follow-up images (Figure 2), and at the end of the tumor removal, the surgeon determined the Simpson resection grade (Table S1). 

Microscopic analysis of DT specimens was performed using standard hematoxylin and eosin (H&E) staining.

### 2.2. Radiological Analysis

DT volume was manually extracted from contrast-enhanced MRI T1 sequences using 3D Slicer software Version 4.8.1 (www.slicer.org) by a manual segmentation [16,17]. All adjacent voxels containing DT were used for the volumetric rendering. Radiomic analysis was performed using the PyRadiomics platform for 3DSlicer [18]. In total, 109 radiomic features were analyzed. The features were classified into First-order statistics (*n* = 21), Gray Level Dependence Matrix (GLDM) (*n* = 14), Shape Descriptors (*n* = 13), Gray Level Co-occurrence Matrix (GLCM) (*n* = 24), Gray Level Run Length Matrix (GLRLM) (*n* = 16), Gray Level Size Zone Matrix (GLSZM) Features (*n* = 16) and Neighboring Gray Tone Difference Matrix (*n* = 5). The features are concisely summed up in Table S2. 

Macroscopic radiological analysis of DT was performed independently by an experienced neuroradiologist and a neurosurgeon. In case of a disagreement, the case was reviewed by both experts and agreed upon. The extent of DT was assessed in terms of the relationship with tumor size and was classified into small, medium and extensive. DT with the extent in at least one direction shorter than 50% of tumor diameter was classified as small. If the extent in one direction was 50–100% of tumor diameter, it was classified as medium. In cases where the extent of DT was larger than 100% of tumor diameter, it was classified as extensive.

### 2.3. Statistics

The histological and radiological DT data were assessed along with clinical information comprising patient age, gender, histology, completeness of tumor removal, and follow-up status. Statistical analysis was performed using SPSS software Version 26.0 (IBM Corporation, Armonk, New York, USA) and RStudio version 1.3.1093 (RStudio, Boston, Massachusetts, USA). Hierarchical clustering of radiomic characteristics was performed using the Ward linkage method and squared Euclidean distance measure; the variables were normalized using z-scores. The association between dural infiltration and patient clusters, the extent of the dural tail, and patient characteristics were assessed by Chi-Square. No correction for multiple testing was performed. *p* < 0.05 was considered statistically significant.

## 3. Results

### 3.1. Patient Characteristics and Histological Analysis

Twenty-two patients were included in the study; 20 (91%) were female. The mean age was 54.2 (Standard deviation (SD) 13.9, range 30–85) years, 16 patients were recruited consecutively between 2015–2017. Main patient characteristics are provided in Table 1. Six patients (27%) had atypical (WHO grade II) meningiomas; the remaining patients were diagnosed with WHO grade I meningiomas. En-bloc resection of the DT with the tumor could be achieved in 4 patients. The remaining 18 patients received piecemeal resection; therefore 72 DT samples from these patients were taken as shown in Figure 1 for further analysis. 

Simpson grade I resection was achieved in 20 (91%) cases and was not possible in 2 (11.1%) patients due to tumor infiltration of the superior sagittal sinus. Respectively, the two medial dura samples after piecemeal resection were infiltrated with tumor cells and were omitted from further analysis. None of the four patients who received en-bloc resection demonstrated dural infiltration on the resection margin. From the patients with piecemeal resection, tumor cell infiltration was detected at the resection margin in 4 of 70 (5.7%) DT samples. The presence of tumor cells could not be excluded in another 5 of 70 (7.1%) samples because of the proliferation of arachnoid cap (meningothelial) cells in the histological specimen that could not be differentiated from tumor cells.

The mean follow-up period was 27.5 (SD 13.2, range 0 to 50.0) months. Two patients died of undisclosed causes before the first follow-up, and four patients were lost to follow-up. No tumor recurrences were detected at follow-up MRI examinations in the remaining 18 patients. There was no significant association between tumor cell infiltration and histological subtype, patient characteristics, or tumor size or shape.

### 3.2. Radiological Analysis of the Dural Tail

**Figure 3:**
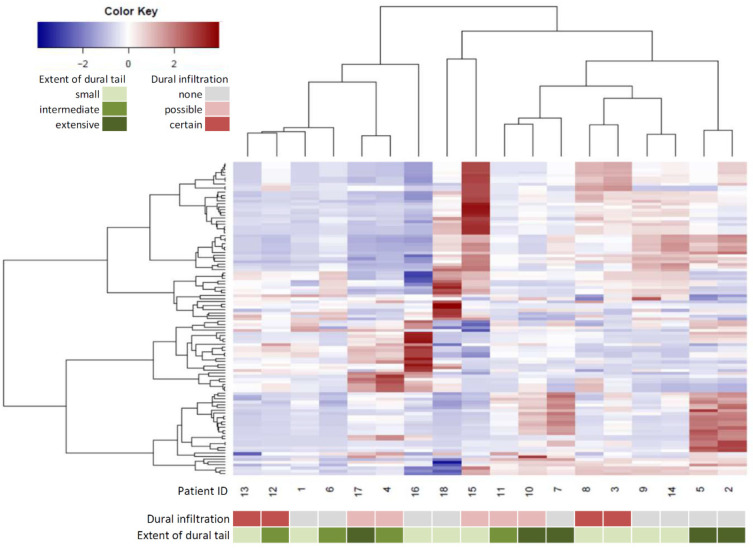
Hierarchical clustering of patients (horizontal axis) according to radiomic features (vertical axis) of the dural tail. The color key of the heat map represents standardized values according to the z-score. Annotation bars represent histologically assessed dural infiltration with tumor cells and the extension of the dural tail.

Preoperative MRI data used for assessment of radiomic features were available for 18 (82%) patients. After extraction of radiomic features, a hierarchical clustering model was generated (Figure 3). Three clusters of patients were identified. Patient number 8 was assigned to the group of “dural infiltration” for this analysis, as the DT segmentation included tumor infiltration in the superior sagittal sinus. No association between clusters and dural infiltration was found *p* = 0.77. The difference between patient clusters remained statistically insignificant, even if the cases with possible infiltration were included (*p* = 0.61).

In 5 patients (28%), an extensive DT was identified, in 4 patients (22%) an intermediate extension, and 9 (50%) showed a small DT (Figure 3). There was no correlation between the extent of DT and tumor infiltration (*p* = 0.39) or extension of DT and radiomic clusters (*p* = 0.89).

## 4. Discussion

Simpson grade I resection of meningiomas requires removal of the dural attachment of the tumor [15]. However, Simpson grading was established before introducing MRI, and its significance in current practice has been questioned [19]. While most authors agree that Simpson grading maintains its relevance [13,20], there is some discrepancy between macroscopically altered dura described by Simpson, the DT on MRI, and histological findings. 

In this series of 22 convexity meningioma patients, we found from targeted sampling at the distant meningioma margin of DT, that at least 8.1% of distal DT samples contained meningioma cells, and tumor cell infiltration could not be excluded in another 6.5%. 

This is in accordance with previously published data showing that tumor cell infiltration in the DT is quite common and may be found in more than two-thirds of all specimens [12]. The extent of tumor cell invasion to the dural tail can range from 1 mm to more than 25 mm from the tumor margin [10,11]. Moreover, a tumor cell infiltration was observed in non-enhancing dura adjacent to meningioma in >30% of cases [12]. The discrepancy between DT sign and tumor infiltration was reported in other tumor entities as well. In a series of 7 operated superficial malignant intracranial tumors with DT sign, leptomeningeal tumor invasion did not correspond to the prominent dural enhancement and was completely absent in 2 cases [21].

As our results demonstrate, tumor cells are commonly found in the outermost part of the DT. This raises the question of what should be accounted as complete tumor resection. Contrast enhancement in distal parts of the DT is probably caused by venous congestion [8]. As such, the limits of this phenomenon might be therefore more dependent on blood flow and less on infiltration by individual tumor cells. 

Tumor infiltration could not be entirely excluded in 4 of 70 excised DT samples in our study. Another four samples showed findings that could not be clearly differentiated into tumor cell infiltration or proliferation of arachnoidal cap cells. Meningothelial hyperplasia is a reactive process characterized by a proliferation of arachnoidal cap cells that can hardly be differentiated from a meningioma cell [22]. For example, it was reported on a hematoma capsule distant from the dura in a patient presenting with a chronic subdural hematoma and meningioma [23]. It is not clear how such a proliferation, which cannot be differentiated from the tumor cell infiltration, should be interpreted when found in the vicinity of a meningioma, and it might be that it is sometimes reported as tumor manifestations. 

Radiomic analysis, based on mathematic processing of imaging data, is a promising tool for diagnostic and treatment planning of meningiomas. It could be used to predict meningioma grade, risk of relapse, and overall survival [24], or used for differential diagnosis between hemangiopericytoma and meningioma [25]. Our attempt to identify the radiomic or macroscopic characteristics of DT associated with the infiltration of tumor cells was unsuccessful. Several technical limitations have to be considered when applying radiomic analysis for dura mater. Firstly, the contrast-enhancing dura can be thinner than one voxel that is required for object segmentation. Secondly, DT might be unevenly distributed around the tumor; therefore, several inhomogeneous volumes have to be analyzed separately. Nevertheless, there can be variations of the radiomic data due to the MRI specifications and interobserver variability. However, the macroscopic evaluation of the DT could not clearly identify tumor-infiltrated DT in this study.

In comparison to macroscopically altered dura mater, the extent of the DT may be far-reaching. The DT may involve up to 3 cm of surrounding dura [6]. To accomplish a complete resection of the DT, the craniotomy should be approximately 1 cm larger than the DT, prompting a significantly larger craniotomy in comparison to conventional meningioma resection [11], consequently requiring a larger skin incision, a more extensive dural reconstruction flap, and more prolonged operation and brain exposure times, leading to an increased risk of surgical morbidity not related to the tumor itself [26]. According to long term follow-up data from Sweden, the recurrence rate of falx meningiomas after Simpson grade I–II resections between 1975 and 1979 (i.e., before the MRI era) was 13% after ten years and 38% after 25 years [27], and the local recurrence rate was approximately 24 to 60% after 15 years after Simpson grade I to III resections [28]. There are as yet no data on whether better long-term results can be achieved after more extensive excision of the surrounding dura. 

Our study suffers from several limitations that must be considered. It is a single-center study with a small partially inconsecutive patient sample and a short follow-up period. Therefore the association of dural infiltration and meningioma recurrence cannot be adequately assessed. It relies on the precision of the navigation system to localize the extent of DT, which may be inaccurate in certain cases. The completeness of tumor and DT resection was assessed by the surgeon, which is subject to bias. Moreover, further histological work-up of suspected cases of DT infiltration was not performed. However, in our opinion, these shortcomings do not compromise the main outcomes of this study. 

There is no clear correlation between tumor infiltration and the extent of the DT. As tumor cells can be found in the distal reaches of DT or even beyond this radiological phenomenon, the extent of meningioma resection should not be guided by a preoperative radiological appearance, especially in cases where the excision of the DT would require a significantly larger craniotomy.

## 5. Conclusions

The radiological dural tail does not reliably outline the extent of tumor cell infiltration in convexity meningiomas. Hence the extent of dural tail resection should not exclusively be guided by the preoperative radiological appearance.

## Figures and Tables

**Table 1:** Patient and tumor characteristics of the study population.

Patient ID	Gender	Age	Simpson Grade	Histology	Dural Tail Infiltration	Follow Up (Months)	Follow-Up Status
1	w	45	1	transitional	No	45.54	no tumor
2	w	52	1	transitional	No	18.60	no tumor
3	m	68	2	angiomatous	2 of 3 *	1.81	dead
4	w	62	1	atypical	Possible 1 of 4	34.00	no tumor
5	w	54	1	secretory	No	10.15	
6	w	36	1	fibrous	No	28.88	no tumor
7	w	44	1	transitional	No	30.52	no tumor
8	w	85	2	fibrous	No *	38.87	deceased
9	w	62	1	transitional	No	37.65	no tumor
10	w	60	1	secretory	Possible 1 of 4	27.14	no tumor
11	w	47	1	transitional	Possible 1 of 4		no tumor
12	w	58	1	atypical	1 of 4	17.91	no tumor
13	w	70	1	meningothelial	1 of 4	23.16	no tumor
14	w	65	1	psammomatous	No	42.61	no tumor
15	w	48	1	atypical	Possible 1 of 4	50.00	no tumor
16	w	30	1	transitional	No	21.59	
17	w	51	1	transitional	Possible 1 of 4		
18	w	55	1	psammomatous	No	28.91	
19	m	31	1	atypical	No	6.77	no tumor
20	w	39	1	atypical	No	31.01	no tumor
21	w	56	1	transitional	No	45.54	no tumor
22	w	74	1	atypical	No	18.60	no tumor

* Simpson grade 2 resection was performed due to infiltration of the superior sagittal sinus. m—male, w—female.

## Data Availability

The data presented in this study are available on request from the corresponding author. The data are not publicly available due to privacy restrictions.

## Supplementary Materials

The following are available online at https://www.mdpi.com/2077-0383/10/6/1177/s1. Table S1: Classification of meningioma resection according to Simpson grade [15], Table S2: Characteristics used for the radiomic analysis of the dural tail.
