# Supplementary material for: Evaluation of Resection Margin after Image-Guided Dural Tail Resection in Convexity Meningiomas
Source: J Clin Med. 2021 Mar 11;10(6):1177. doi: 10.3390/jcm10061177 (PMC8000745; doi:10.3390/jcm10061177)
Supplement: Supplementary file 1 [file jcm-10-01177-s001.pdf]

Supplemental Table S1. Classification of meningioma resection according to Simpson grade†

Resection grade	Definition
1	Complete resection of tumor, dural attachment and abnormal bone
2	Complete resection of tumor, coagulation of dural attachment
3	Complete resection of tumor without resection or coagulation of dural attachment or resection of extradural tumor extention
4	Partial tumor resection, leaving intradural tumor in situ
5	Simple decompression (biopsy)

†Simpson, D. The recurrence of intracranial meningiomas after surgical treatment. J. Neurol. Neurosurg. Psychiatry 1957, 20, 22–39, doi:10.1136/jnnp.20.1.22.

Supplemental Table S2. Characteristics used for the radiomic analysis of the dural tail

**Intensity**	**Texture**	**Gray Level Run Length Matrix**
Interquartile Range	**Gray level dependence matrix**	Short Run Low Gray Level Emphasis
Skewness	Gray Level Variance	Gray Level Variance
Uniformity	High Gray Level Emphasis	Low Gray Level Run Emphasis
Median	Dependence Entropy	Gray Level Non Uniformity Normalized
Energy	Dependence Non Uniformity	Run Variance
Robust Mean Absolute Deviation	Gray Level Non Uniformity	Gray Level Non Uniformity
Mean Absolute Deviation	Small Dependence Emphasis	Long Run Emphasis
Total Energy	Small Dependence High Gray Level Emphasis	Short Run High Gray Level Emphasis
Maximum	Dependence Non Uniformity Normalized	Run Length Non Uniformity
Root Mean Squared	Large Dependence Emphasis	Short Run Emphasis
90th Percentile	Large Dependence Low Gray Level Emphasis	Long Run High Gray Level Emphasis
Minimum	Dependence Variance	Run Percentage
Entropy	Large Dependence High Gray Level Emphasis	Long Run Low Gray Level Emphasis
Range	Small Dependence Low Gray Level Emphasis	Run Entropy
Variance	Low Gray Level Emphasis	High Gray Level Run Emphasis
10th Percentile	**Gray level co-occurrence matrix**	Run Length Non Uniformity Normalized
Kurtosis	Joint Average	**Gray Level Size Zone**
Mean	Sum Average	Gray Level Variance
**Shape**	Joint Entropy	Zone Variance
Maximum 3D Diameter	Cluster Shade	Gray Level Non Uniformity Normalized
Maximum 2D Diameter Slice	Maximum Probability	Size Zone Non Uniformity Normalized
Sphericity	IDMN	Size Zone Non Uniformity
Minor Axis	Joint Energy	Gray Level Non Uniformity
Elongation	Contrast	Large Area Emphasis
Surface Volume Ratio	Difference Entropy	Small Area High Gray Level Emphasis
Volume	Inverse Variance	Zone Percentage
Major Axis	Difference Variance	Large Area Low Gray Level Emphasis
Surface Area	IDN	Large Area High Gray Level Emphasis
Flatness	IDM	High Gray Level Zone Emphasis
Least Axis	Correlation	Small Area Emphasis
Maximum 2D Diameter Column	Autocorrelation	Low Gray Level Zone Emphasis
Maximum 2D Diameter Row	Sum Entropy	Zone Entropy
	MCC	Small Area Low Gray Level Emphasis
	Sum Squares	**Neighbouring Gray Tone Difference Matrix**
	Cluster Prominence	Coarseness
	IMC2	Complexity
	IMC1	Strength
	Difference Average	Contrast
	ID	Busyness
	Cluster Tendency	
